# Recent Advances in Assessing Immunogenicity of Therapeutic Proteins: Impact on Biotherapeutic Development

**DOI:** 10.1155/2016/8141269

**Published:** 2016-08-23

**Authors:** Yanmei Lu, Leslie A. Khawli, Shobha Purushothama, Frank-Peter Theil, Michael A. Partridge

**Affiliations:** ^1^Departments of Biochemical & Cellular Pharmacology, Genentech, Inc., South San Francisco, CA 94080, USA; ^2^Keck School of Medicine, University of Southern California, Los Angeles, CA 90033, USA; ^3^UCB Pharma, Slough, Berkshire SL1 14EN, UK; ^4^Regeneron Pharmaceuticals, Inc., Tarrytown, NY 10591, USA

Biologics such as monoclonal antibodies, recombinant proteins, and novel protein scaffolds can elicit an unwanted immune response in patients. This response may produce neutralizing and/or nonneutralizing antidrug antibodies (ADA) that can impact drug pharmacokinetics, clinical efficacy, and patient safety. Therefore, it is critical to detect an immunogenic response and characterize ADA in both preclinical and clinical phases of development.

The special issue provides a snapshot of some of the ongoing efforts in the area of immunogenicity such as the prediction, detection, and characterization of the ADA responses as well as the emerging area of understanding how to deal with preexisting ADA. Although the topics adopted by the different papers are diverse, the common theme that unifies these papers is the need to solve the fundamental problems of immunogenicity by using various strategies. Some of the problems addressed by the papers in this thematic issue have a long history, such as the need to better understand the impact of immunogenicity on study outcomes, the corelation of ADA incidence to clinical relevance, and the need to improve our knowledge of manufacturing processes that impact immunogenicity.

M. A. Partridge et al. review the use of technologies such as SQI SquidLite, Genalyte Maverick System, and immunocapture-LC/MS for simultaneous detection and isotyping of ADA response. The pros and cons of using immune PCR for improved drug tolerance and sensitivity as well as the Gyrolab for decreased reagent use and automated workflow are also discussed. Selection of the appropriate technology platform (ELISA, Meso Scale Discovery, Gyrolab, and AlphaLISA) for improved assay sensitivity and drug/soluble target tolerance to reduce false positive rate has been discussed by J. Collet-Brose et al. The improvement in assay sensitivity and drug/soluble target tolerance allows the identification of a much greater ADA positive rate. S. Song et al. present their perspective on correlating unexpected high ADA incidence with clinical relevance to provide physicians with clinically meaningful immunogenicity results. Moreover, different approaches to the use of a generic ADA assay in preclinical testing are presented by M. Carrasco-Triguero et al. and M. Boysen et al. The merits of generic ADA assays include minimal assay development time, in part because these methods do not require reagents specific to the therapeutic molecule. These advantages may significantly advance the drug discovery timeline. A timely paper by J. Ruppel et al. discusses how to deal with the challenge of preexisting ADA to the hinge region of F(ab′)2 therapeutic molecule. The preexisting ADA levels vary considerably between animals, necessitating the use of an individual cut-point for each animal in order to detect treatment induced ADA. Lastly, R. J. Kubiak et al. present evidence that conjugated reagents formulated and stored in a histidine-sucrose buffer had superior assay performance compared with reagents in PBS.

Fully human therapeutic monoclonal antibodies have been developed in part to lower immunogenicity. Although making the antibody sequence more “self” reduces ADA incidence, clinical data has shown that even “fully” human monoclonal antibodies can induce an antibody response. Knowledge accumulated over the years indicates that many other factors can affect the immunogenicity in addition to the antibody sequence. Therefore, it is important to review the underlying mechanism and identify critical contributing factors of antibody response. In this special issue, A. Kuriakose et al. discuss different causes of antibody responses that are related to inherent properties of the therapeutic molecule, processes in manufacturing the product, patient characteristics, and route of administration. This and another review article in the special issue (R. Jefferis et al.) elaborate on the association of posttranslational modification and immunogenicity.

In addition, a review paper by A. Smith et al. describes the enhanced understanding of the impact of immunogenicity on study outcomes. Specifically, the paper touched upon different preclinical in silico, in vitro, and in vivo tools for immunogenicity risk assessment and the effect of immunogenicity on PK/PD, efficacy, and safety of large molecule therapeutics.

Biotherapeutics have evolved from antibodies to novel modalities such as multidomain proteins, multispecific antibodies, nanobodies, and the like. The collection of papers in this thematic issue covers a range of topics in the field of immunogenicity, including understanding the causes of ADA development, predictive immunogenicity before administration in humans, mitigating interference in ADA assays, and examination of new technologies in ADA detection.

